# Oxidative stress and ROS-mediated cellular events in RSV infection: potential protective roles of antioxidants

**DOI:** 10.1186/s12985-023-02194-w

**Published:** 2023-10-05

**Authors:** Xue Yang, Xue Liu, Yujun Nie, Fei Zhan, Bin Zhu

**Affiliations:** Department of Pediatrics, Xiangyang Central Hospital, Affiliated Hospital of Hubei University of Arts and Science, Xiangyang, 441021 Hubei China

**Keywords:** Respiratory syncytial virus, Oxidative stress, Reactive oxygen species, Nrf2, NLRP3, NETs, HMGB1

## Abstract

Respiratory syncytial virus (RSV), a member of the Pneumoviridae family, can cause severe acute lower respiratory tract infection in infants, young children, immunocompromised individuals and elderly people. RSV is associated with an augmented innate immune response, enhanced secretion of inflammatory cytokines, and necrosis of infected cells. Oxidative stress, which is mainly characterized as an imbalance in the production of reactive oxygen species (ROS) and antioxidant responses, interacts with all the pathophysiologic processes above and is receiving increasing attention in RSV infection. A gradual accumulation of evidence indicates that ROS overproduction plays an important role in the pathogenesis of severe RSV infection and serves as a major factor in pulmonary inflammation and tissue damage. Thus, antioxidants seem to be an effective treatment for severe RSV infection. This article mainly reviews the information on oxidative stress and ROS-mediated cellular events during RSV infection for the first time.

## Introduction

Respiratory syncytial virus (RSV), which belongs to Orthopneumovirus genus of the Pneumoviridae family, is a frequent pathogenic agent responsible for respiratory infections in infants, young children, the elderly and people with poor immune function [1–3]. The single-stranded RNA genome is enclosed in a lipoprotein envelope with major transmembrane proteins, including the viral attachment protein (G) and fusion protein (F) which contribute to the infection of host cells. RSV significantly disrupts the airway and alveolar epithelium and induces airway inflammation with neutrophil infiltration, accumulation of mucus, cellular debris, and edema, leading to airway obstruction and turbulent gas flow [4]. Afterward, RSV infection may give rise to long-term sequelae such as repeated wheezing and asthma, which result in increased health care costs and reduced quality of life [3, 4]. In addition, since infection with RSV does not lead to long-term immunity, children and adults can suffer from recurrent respiratory infections. A recent systematic analysis revealed that RSV is a significant cause of morbidity and mortality in children aged 0 to 5 years around the world, particularly in the first six months of life and in low-income and middle-income countries [2]. Additionally, with more physical encounters brought on by the relaxation of COVID-19 restriction measures, RSV-related hospitalizations among children under the age of five can be anticipated to rise, potentially exceeding the numbers reported in prior RSV seasons [5]. The real-world disease burden caused by RSV makes it an urgent matter to develop effective antivirals and vaccines for RSV. Fortunately, recent advancements have been made. RSV prefusion F protein-based (RSVpreF) vaccine (Abrysvo, Pfizer), RSVPreF3 vaccine (Arexvy, GSK), and mRNA-1345 (Moderna) have been approved by the US Food and Drug Administration (FDA) for the prevention of RSV infection in people over 60 years of age recently [6, 7]. Moreover, the first maternal RSVpreF vaccine (Abrysvo, Pfizer) was recently greenlit by the US FDA to prevent severe illness in infants from birth to 6 months [8]. Monoclonal antibody (mAb) prophylaxis is another prevention strategy against RSV infections in a small subset of very high-risk infants and young children, but is expensive for the low-middle income settings. Palivizumab, a monoclonal anti-RSV fusion protein, is the first antiviral mAb approved for RSV treatment. Even so, it is currently approved only for the prophylactic treatment of RSV in specific infants due to its high costs and limited effectiveness [9]. Nirsevimab is a novel long-acting mAb product intended for use in newborns and infants to protect against RSV disease that was approved by the FDA recently. But Nirsevimab’s cost will still be a potential implementation barrier, particularly for ambulatory practices [10]. Therefore, it is necessary and pressing to develop affordable and effective interventions through a better understanding of cellular factors and signaling events that regulate RSV infection.

A clinical study determined that oxidative stress may contribute to the pathogenesis of RSV-induced acute bronchiolitis and correlate with the disease severity [11]. The definition of oxidative stress indicates “an imbalance between pro-oxidants and antioxidants with concomitant dysregulation of redox circuits and macromolecular damage” [12]. Oxidative stress is often recognized as a signaling mechanism for many cellular processes, including proliferation, differentiation, senescence, signaling, transcription factor activation, apoptosis, motility, and metabolism [13]. As pro-oxidants, reactive oxygen species (ROS) comprise superoxide anion (O_2_^−^), hydroxyl radical (HO^·^), hydrogen peroxide (H_2_O_2_), and some other reactive molecules and free radicals. Cellular ROS are attributed to mitochondrial oxidative metabolism or ROS-generating enzymes, mainly nicotinamide adenine dinucleotide phosphate oxidases (NADPH oxidases, NOX) and xanthine oxidase (XO). Antioxidant enzymes (AOEs) include superoxide dismutase 1 (SOD 1), SOD 2, SOD 3, catalase, glutathione peroxidase, and glutathione S-transferase, which can either directly decompose ROS or facilitate antioxidant reactions. Accumulated evidence has shown that ROS are essential intracellular second messengers that work in complex signaling networks and regulate various cellular signaling processes [14]. Many lines of evidence have shown that marked signs of increased production of ROS accompany multiple respiratory viral infections, including RSV, influenza A viruses, Sendai virus, severe acute respiratory syndrome coronavirus 2 (SARS-CoV-2), and rhinovirus [15–19], and the serum level of O_2_^−^ is a predictor of COVID-19 severity in patients [20]. ROS are known to contribute to the suppression of some respiratory infections through the induction of innate immune responses [21]. However, excessive ROS can induce cell death, which boosts the release and spread of virions and thus stimulates the replication of respiratory viruses with a lytic life cycle [22]. Furthermore, overproduction of ROS plays an important role in cellular events including Nod-like receptor protein (NLRP) 3 inflammasome activation, neutrophil extracellular trap (NET) and high-mobility group box 1 (HMGB1) release, DNA damage and acquired ciliopathies, which contribute to severe RSV disease. Thus, antioxidant treatments seem to be effective in ameliorating severe RSV infection [15].

RSV infections and ROS production, oxidative stress, viral replication, and the immune response are related. Therefore, this review mainly analyzes the key roles of ROS and information on the oxidative stress associated with the infections caused by RSV.

## Sources of ROS in RSV-infected cells

NADPH oxidases, as ROS-generating enzymes, catalyze the production of the superoxide anion via one-electron transmembrane transfer to molecular oxygen. There are seven NADPH oxidase isoforms: NOX1-NOX5, DUOX1, and DUOX2 [23, 24]. These NADPH oxidase isoforms are expressed in different membranes depending on the cell type. They are distributed in the phagosome, mitochondria, endoplasmic reticulum, nucleus and plasma membrane for ROS production [25–27]. RSV can induce ROS generation by neutrophils, and pretreatment with the NADPH oxidase inhibitor diphenyleneiodonium (DPI) abrogates this effect, indicating that RSV stimulates ROS generation through NADPH oxidase activation to a certain degree [28]. Among the NADPH oxidases, NOX2 is classically expressed in cells of the innate immune system, including neutrophils, monocytes, and macrophages [29–31]. Notably, NOX2 is expressed in epithelial cells to a lesser extent. ROS have been shown to act as a redox switch required for efficient RSV-mediated activation of the nuclear factor kappa-light-chain-enhancer of activated B cells (NF-κB) and interferon regulatory factor (IRF) 3 activation pathways [32, 33]. NOX2 has been identified as a specific source of ROS responsible for the oxidant-dependent activation of NF-κB observed in RSV-infected human airway epithelial cells (hAECs) [34]. Induction of NOX1 in A549 cells during RSV infection also leads to the upregulation of ROS [35]. Further study is needed to identify the contribution of other ROS-generating enzymes to ROS production in different kinds of cells.

Mitochondria are the main location of aerobic respiration to supply energy and other notable sources of ROS in cells [36, 37]. Generally, various viruses enhance the generation of mitochondrial ROS (mtROS), which activates certain host cellular pathways that facilitate viral replication [38–40]. This can also be confirmed in cells infected by RSV [41, 42]. RSV can induce microtubule/dynein-dependent mitochondrial perinuclear clustering and translocation toward the microtubule-organizing center, which is concomitant with impaired mitochondrial respiration, loss of mitochondrial membrane potential, and increased production of mtROS. Agents that target dynein/microtubules or inhibit mtROS production strongly reduce RSV production and lung inflammation, which indicates that RSV utilizes host cell mitochondria to boost mtROS production to facilitate virus replication [41]. Further study revealed that mitochondrial complex I activity is central to RSV infection. Reduced activity of complex I leads to impaired mitochondrial respiration, to increased mtROS generation and to enhanced virus production, which can be reversed by the mtROS scavenger mitoquinone mesylate (MitoQ) [43]. In addition, RSV induces endoplasmic reticulum (ER) stress and a noncanonical unfolded-protein response (UPR) [44], which triggers ER Ca^2+^ release and transport into mitochondria, leading to mitochondrial lumen Ca^2+^ (mt-Ca^2+^) overloading [45]. As an increased level of mt-Ca^2+^ is a key factor for the production of mtROS [45], we infer that the RSV-induced ER stress/mt-Ca^2+^/mtROS cascade is another pathway of mtROS production in RSV infection. Moreover, increased immune-responsive gene-1 (IRG1) induction in AECs and mouse lungs is responsible for ROS production during RSV infection [46]. IRG1 induction is described to enhance the production of ROS presumably through the promotion of the pentose phosphate pathway [47]. In contrast, itaconate, which is generated by IRG1 in the mitochondrial matrix, works as a fine activator of nuclear factor erythroid 2-related Factor 2 (Nrf2) [46, 48]. Thus, RSV-induced IRG1 generation seems to play a complicated role in oxidative stress.

## Oxidative stress and enhanced ROS production during RSV infection

**Fig. 1:**
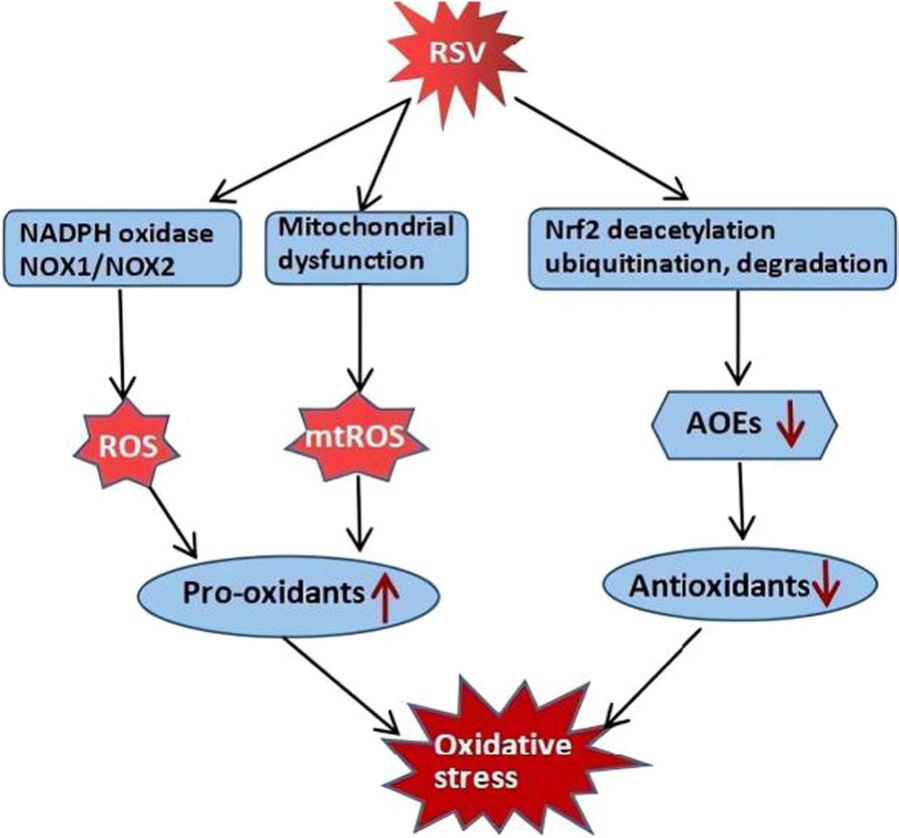
Oxidative stress and sources of ROS in RSV infection. To the best of our knowledge, RSV-induced ROS production is partially attributed to NADPH oxidases/NOX, among which NOX1 and NOX2 are involved in ROS generation by epithelial cells. RSV can induce mitochondrial ROS (mtROS) via mitochondrial respiration damage and dysfunction. RSV can also promote Nrf2 deacetylation, ubiquitination, and degradation, leading to decreased expression of AOEs. All of these cellular events result in an imbalance between pro-oxidants and antioxidants, leading to oxidative stress

RSV infection is associated with increased production of ROS, degradation of Nrf2, and decreased expression of AOEs in cells, mice, and children, leading to oxidative stress and lung damage (Fig. 1) [15, 49–53]. ROS are byproducts of cellular metabolism and can be either beneficial, at low levels, or harmful, at high levels, to the cell. However, in experimental animal or cell models of RSV infection, an increase in ROS levels and the existence of oxidative stress have been demonstrated, and inhibiting ROS generation was shown to improve the disease, which indicates the unfavourable role that oxidative stress and ROS play in RSV infection [15, 28, 35, 46, 54]. Enhanced ROS production can be observed in the human airway epithelial cell lines BEAS-2B and A549 and in immune cells such as neutrophils and macrophages during RSV infection [15, 28, 35, 54].

Nrf2 is an important redox-responsive protein and the master regulator of the expression of AOEs, which helps protect cells from oxidative stress and injury [55]. Under normal physiological conditions, most Nrf2 is sequestered in the cytoplasm by its covalently bound inhibitor Kelch-like ECH-associated protein 1 (Keap1) and cannot translocate into the nucleus [56–59]. Under oxidative stress, Nrf2 detaches from Keap1 and translocates to the nucleus, where it heterodimerizes with one of the small Maf (musculoaponeurotic fibrosarcoma oncogene homolog) proteins, which facilitates its binding to the antioxidant response elements (AREs) and activates ARE-dependent gene expression of a series of antioxidative, antidotal and cytoprotective proteins [57]. However, during infection, RSV induces ROS overproduction but Nrf2 deacetylation, ubiquitination, and degradation via the proteasome pathway both in vitro and in vivo, which leads to decreased expression of ARE [53, 60]. Upregulating the expression of Nrf2 and AOEs can reduce RSV infection in human lung epithelial cells and mitigate RSV-induced injury and oxidative stress in mice [53, 61]. A recent study indicated that the H_2_S donor GYY4137 decreases Keap1 levels and modulates pathways involved in RSV-induced Nrf2 degradation, contributeing to the significantly increased Nrf2 levels and AOE gene expression, which could be explored as a potential therapeutic application in RSV infection [62].

## ROS-mediated cellular events in RSV infection

### ROS and NLRP3 inflammasome activation

**Fig. 2:**
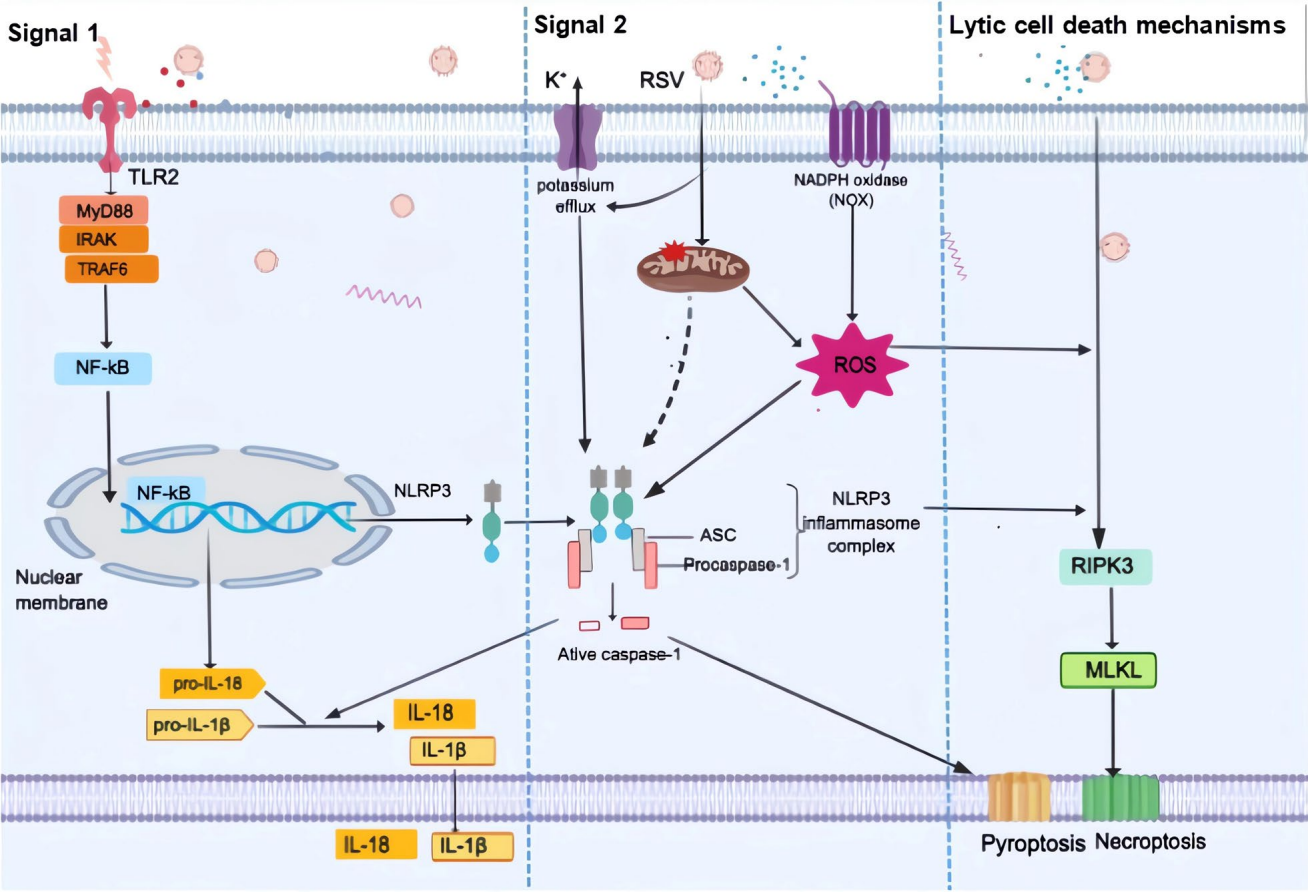
ROS-mediated NLRP3 inflammasome activation and lytic cell death in RSV-infected macrophages. Signal 1: Activation of the TLR/Myd88/NF-κB pathway promotes the expression of NLRP3,pro-IL-1β, and pro-IL-18. Signal 2: RSV-induced ROS overproduction and potassium (K^+^) efflux as well as mitochondrial dysfunction contribute to NLRP3 inflammasome activation, leading to caspase 1 activation, which induces the cleavage of pro-IL-1β and pro-IL-18 into their mature and biologically active forms. Lytic cell death mechanisms: ASC-NLRP3 inflammasome-dependent caspase-1 activation promotes pyroptotic cell death and may also partially contribute to activation of the necroptosis pathway in RSV-infected macrophages during RSV infection. The RIPK3/MLKL pathway plays a positive regulatory role in necroptosis. The pivotal role of ROS in promoting lytic cell death in RSV-infected macrophages is also highlighted

As germline-encoded receptors in host cells, pattern recognition receptors (PRRs), which include Toll-like receptors (TLRs), C-type lectin receptors (CLRs), retinoic acid-inducible gene-I (RIG-I)-like receptors (RLRs), and Nod-like receptors (NLRs), recognize and respond to diverse pathogen-associated molecular patterns (PAMPs) and damage-associated molecular patterns (DAMPs) derived from invading viruses, thus becoming involved in the innate immune system [63]. PRRs can act as cytosolic PAMP and DAMP sensors and form the protein complexes known as inflammasomes, among which the NLRP3 inflammasome is the most well studied and plays a vital role in both inflammation and host antiviral immune responses.The three major components of the NLRP3 inflammasome are NLRP3, which captures the danger signals and recruits downstream molecules; procaspase-1, which elicits maturation of the cytokines interleukin (IL)-1β and IL-18 and processing of gasdermin D to mediate cytokine release and pyroptosis; and apoptosis-associated speck-like protein containing a caspase recruitment domain (ASC), which functions as a bridge connecting NLRP3 and procaspase-1 [64]. Activation of the NLRP3 inflammasome requires two signals during viral infections (Fig. 2). The first is signal 1 (priming signal); the activation of PRRs, tumor necrosis factor receptors (TNFRs), or interferon receptors (IFNRs) induces NF-κB activation and triggers the transcription of NLRP3, pro-caspase-1, pro-IL-1β, and pro-IL-18. The second is signal 2 (activation signal); multiple DAMPs and PAMPs induce NLRP3 inflammasome assembly and activation [65]. Currently, NLRP3 stimuli have been demonstrated to induce multiple molecular and cellular signaling events, including ionic flux, mitochondrial dysfunction, the generation of ROS (especially mtROS), and lysosomal damage, leading to the activation of the NLRP3 inflammasome [66, 67]. Among the “second signals”, the ROS model represents a common pathway underlying NLRP3 inflammasome activation [68] since ROS are at the crossroads of inflammasome and inflammation [69]. Overproduction of ROS is reported to be essential and is involved in diverse signal axes for NLRP3 inflammasome activation [70–72]. ROS-thioredoxin-interacting protein (TXNIP)-NLRP3 axis activation is responsible for various pathogeny-induced organ or tissue injuries [73–75]. Moreover, ROS can activate NLRP3 by inducing Ca^2+^ influx through transient receptor potential melastatin 2 (TRPM2) channels [73]. ROS can also serve as an important inflammasome-activating signal and activate inflammasomes through mitogen-activated protein kinases (MAPK) and extracellular signal-regulated protein kinases 1 and 2 (ERK1/2) [69]. Furthermore, NLRP3 inflammasome-driven inflammation recruits inflammatory cells like neutrophils and macrophages, which in turn produce ROS, indicating a feedback loop between ROS and NLRP3 inflammasome [67].

Many lines of evidence suggest that RSV can activate the NLRP3 inflammasome and induce inflammasome-related airway inflammation, even pyroptosis [70, 76, 77], similar to other RNA viruses [73]. As we mentioned before, RSV can induce excessive production of ROS [15, 54], which contributes to the activation of the NLRP3 inflammasome in RSV infection (Fig. 2) [70, 78]. An earlier cell study illustrated that the priming signal is exerted by activation of the TLR2/myeloid differentiation primary response 88 (MyD88)/NF-κB pathway, which is required for pro-IL-1β and NLRP3 gene expression during RSV infection [78]. In fact, except for TLR3, all TLRs recruit MyD88 to their receptor complex, as do members of the IL-1 receptor family. MyD88 recruits interleukin-1 receptor-associated kinase 1 (IRAK1), IRAK4, and then TNF receptor-associated Factor 6 (TRAF6), which results in the activation of NF-κB [79]. Afterward, as “second signals”, intracellular ROS and potassium (K^+^) efflux due to stimulation of ATP-sensitive ion channels are involved in triggering inflammasome activation [78], leading to caspase 1 activation and turning pro-IL-1β and pro-IL-18 into their mature and biologically active forms [78, 80]. Lytic cell death is an inflammatory cell death caused primarily by three distinct cellular mechanisms: pyroptosis, necroptosis, and ferroptosis [81–83]. Since intracellular constituents released from dying cells are among the stimuli that trigger proinflammatory gene expression programs in innate immune cells, inhibition of cell death may work as a potential therapeutic strategy for excessive or chronic inflammation [84]. A recent study revealed that RSV induces lytic cell death mechanisms in macrophages, specifically through ASC-NLRP3 inflammasome activation of both caspase-1-dependent pyroptosis and receptor-interacting serine/threonine-protein kinase 3 (RIPK3), as well as mixed lineage kinase domain-like pseudokinase (MLKL)-dependent necroptosis [70]. The ASC-NLRP3 inflammasome may also partially contribute to the activation of the necroptosis pathway during RSV infection [70]. A critical role of ROS is underlined in positively regulating lytic cell death of RSV-infected macrophages, and inhibition of ROS by DPI, a potent ROS inhibitor, would dampen lytic cell death [70]. Moreover, NLRP3 inflammasome activation by mtROS plays a significant role in pathogenesis of exaggerated inflammation and targeting mtROS-NLRP3 inflammasome axis may be a promising strategy for alleviating lung injury and treating acute lung injury (ALI) /acute respiratory distress syndrome (ARDS) [85].

Although the NLRP3 inflammasome is a key player in antiviral responses [65], it also leads to an excessive innate immune response, which is implicated in RSV-induced lung inflammation and damage. Multiple interventions and treatments have been reported to attenuate and inhibit the activation of the NLRP3 inflammasome during RSV infection, which abrogates lung inflammatory injury and long-term airway disease development [76, 77, 80, 86]. Thus, it is plausible to infer that inhibiting ROS production may serve as a potential measure to prevent ROS-mediated NLRP3 inflammasome activation and lytic cell death, which may ameliorate RSV-induced lung inflammatory damage. However, the effect of inhibiting ROS-mediated NLRP3 inflammasome activation on RSV replication remains unclear. Further study is still needed.

### ROS and NET release

In addition to the traditional antimicrobial mechanisms of phagocytosis, ROS production, and degranulation, neutrophils can also fabricate NETs, which are important structures comprising a web-like deoxyribonucleic acid (DNA) backbone coated with antimicrobial proteins, such as histones, neutrophil elastase (NE), myeloperoxidase (MPO), and α-defensins, to prevent the spread of infectious virions [87]. NETs arise from the release of granular and nuclear contents of neutrophils in the extracellular space in response to microorganisms, soluble factors, and host molecules. NETosis is a unique and dynamic cell death program for neutrophils that is responsible for NET formation [88]. The specific cell components and signaling cascades required for NETosis to release these DNA threads may vary depending on the stimulus [89]. Delgado-Rizo has reviewed three models for NETosis: ROS-dependent suicidal NETosis, ROS-dependent vital NETosis, and ROS-independent vital NETosis (reviewed in  [90]). Suicidal NETosis, mainly with a duration of 2–4 h, is the principal and best-described model for NET release [91, 92], even though the molecular mechanisms are still not well understood [93]. Once activated through the recognition of stimuli, neutrophils begin to package and activate the NADPH oxidase complex through protein kinase C (PKC)/Raf/MERK/ERK to promote ROS production, which acts as a second messenger in suicidal NETosis by promoting the gradual separation and loss of the nuclear membrane and peptidyl arginase deaminase 4 (PAD4)-mediated histone citrullination, allowing for chromatin decondensation [90, 94]. Increased cytosolic Ca^2+^ also serves as a cofactor for PAD4, which promotes the deamination of histones and allows the decondensation of chromatin, contributing to nuclear DNA release and suicidal NETosis [95]. In vital NETosis, neutrophils release NETs without exhibiting a loss of nuclear or plasma membrane within 5–60 min, and it occurs independently of ROS and the Raf/MERK/ERK pathway [90]. Finally, another type of vital NETosis dependent on ROS has been described, in which mitochondrial DNA is released instead of nuclear DNA [96].

**Fig. 3:**
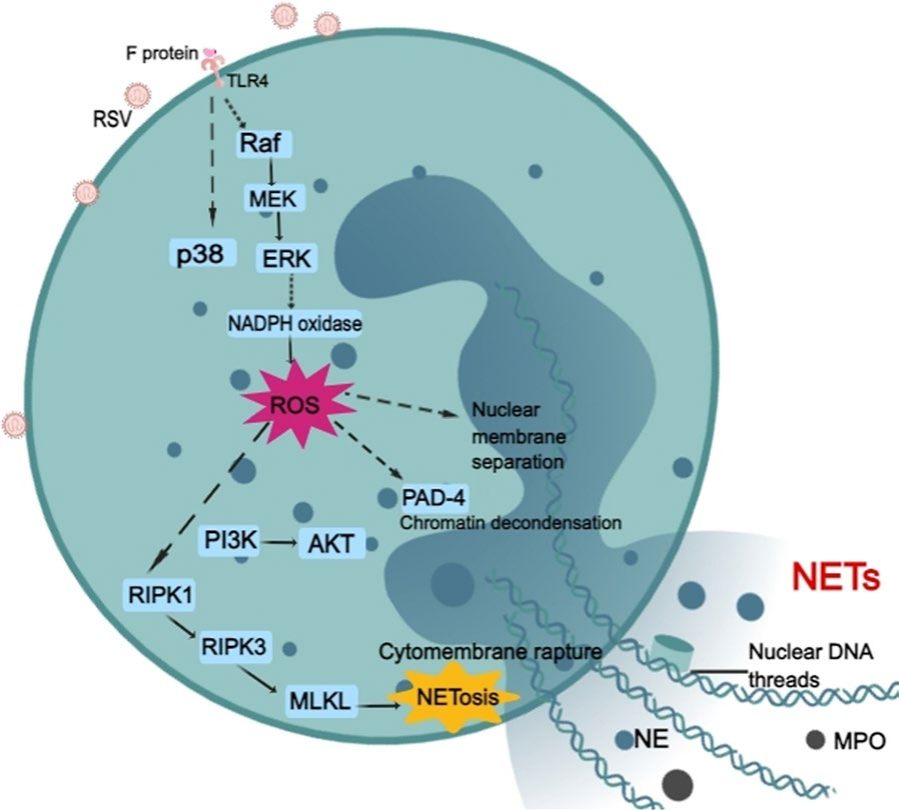
ROS-dependent NET release and NETosis by RSV-infected neutrophils. RSV F glycoprotein stimulates TLR4 which may activate the Raf/MEK/ERK pathway and subsequent NADPH oxidase complexes, leading to ROS production. ROS can promote the separation and loss of the nuclear membrane and activate PAD4-mediated histone citrullination to allow chromatin decondensation. RIPK1/RIPK3/MLKL induces cytomembrane rupture and contributes to NETosis. PI3K/AKT and p38 MAPK are also involved in NETosis, although the mechanism is not fully understood

During RSV infection, an innate immune response characterized by the release of chemokines and cytokines is activated, which promotes neutrophil influx into the respiratory tract [97–99]. The influx of neutrophils to the lungs is associated with increased airway mucus production, cellular inflammation, ROS production, NETosis, and heightened lung damage [100]. Neutrophil-endothelial interactions in RSV bronchiolitis seem to be with a potential for prediction of severity of disease [99]. RSV particles and RSV F protein are both reported to induce NET formation by human neutrophils (Fig. 3). Moreover, RSV F protein is capable of stimulating NET release in a manner dependent on TLR-4 activation, potent NADPH oxidase-derived ROS production, and ERK and p38 MAPK phosphorylation [101]. The enzyme PAD-4-mediated histone citrullination is known to be crucial to chromatin decondensation during NETosis [89]. RSV is able to induce classical ROS-dependent NET release through PAD-4 and necroptosis pathway activation, which is also dependent on the phosphatidylinositol 3-kinase (PI3K)/AKT, ERK, and p38 MAPK pathways. Likewise, RIPK1, RIPK3, and MLKL are essential to RSV-induced NETosis as well, among which MLKL, the necroptosis executioner protein, is likely to promote membrane-disrupting pores leading to neutrophil lysis and NET extrusion. In addition, neutrophils can be stimulated by RSV-infected alveolar epithelial cells or lung fibroblasts and release NETs in a virus replication- and concentration-dependent manner [28]. RSV triggers the formation of NE and MPO, which have also been shown to modulate NET release [102].

The NET formation has been widely demonstrated as an effective mechanism to prevent microbial dissemination and avoid uncontrolled infections. For instance, the presence of NETs decreases RSV-induced cellular damage, possibly by directly affecting viral particle capture and/or interfering with the fusion activity of the F protein [103]. However, NETs are supposed to be a double-edged sword, and excessive NET formation has been described in many human diseases, infectious and noninfectious [104]. For example, excessive NET formation is implicated in the pathogenesis of ventilator-induced lung injury [105], and NETs are also identified as a key marker of disease severity and treatment response in bronchiectasis [106]. Furthermore, although NETs are able to capture RSV and exhibit a potential antiviral effect, they aggravate the immune response of the infection and cause airway obstruction in infants with severe RSV-LRTI [107]. Calves with severe bovine RSV infection also suffer from an extensive release of NETs, which causes airway obstruction [107, 108]. Since NETs are decorated with NE, MPO, and granule proteins, they can induce pathogen-induced lung injury and inflammation [109, 110]. Moreover, the overactivated ROS-NET pathway plays a role in an extensive microthrombus formation with multiorgan failure in systemic viral infection [111], which indicates the harmful effects of excessive NET formation. Treatment with DPI can significantly inhibit F protein-induced NET production in RSV-infected neutrophils [101], and inhibition of ROS production can decrease NET formation, cellular inflammation, mucus hypersecretion and diminish the severity of viral bronchiolitis in mice [100]. These results indicate that RSV tends to induce ROS-dependent suicidal NETosis. Targeting the overproduction of ROS may regulate NET formation and attenuate lung damage and inflammation in severe respiratory virus infection such as RSV. To date, as most researchers consider the adverse side effects of excessive NETs, reducing the formation and release of NETs has received increasing attention. Another strategy to modulate the neutrophil response and NET release is the ligation of inhibitory receptors, also known as immune checkpoints [112, 113]. Through a study of fresh neutrophils from the airways of a large cohort of infants with life-threatening RSV infection and controls, two immune checkpoints, signal inhibitory receptor on leukocytes (SIRL)-1 and leukocyte-associated immunoglobulin-like receptor (LAIR)-1, were found to regulate neutrophil function, inhibit NET formation by sputum neutrophils of RSV patients and modulate the course of severe RSV bronchiolitis in a positive way [113]. Collectively, excessive NETs are considered to do more harm than good in severe RSV infection. Inhibition of overproduction of ROS to reduce the release of NETs seems to be a potential strategy to improve the severe RSV diseases.

### ROS and HMGB1 release

HMGB1 is a ubiquitous redox-sensitive chromatin-binding protein that can be released into the extracellular space to function as a proinflammatory cytokine. As an alarmin protein, HMGB1 is involved in human host defense and immune surveillance by alerting the immune system to infectious and tissue damage signals and triggering an immediate response. However, when misregulated, HMGB1 can become deleterious and induce serious cell and tissue damage [114], which is involved in many pathological conditions, including systemic lupus erythematosus, colorectal cancer, ischemia/reperfusion injury, and type 2 diabetes [115–118]. Several cellular mechanisms, such as vesicular transport, inflammasome activation, ROS generation, and necroptosis, are known to regulate HMGB1 release [97, 119–121].

**Fig. 4:**
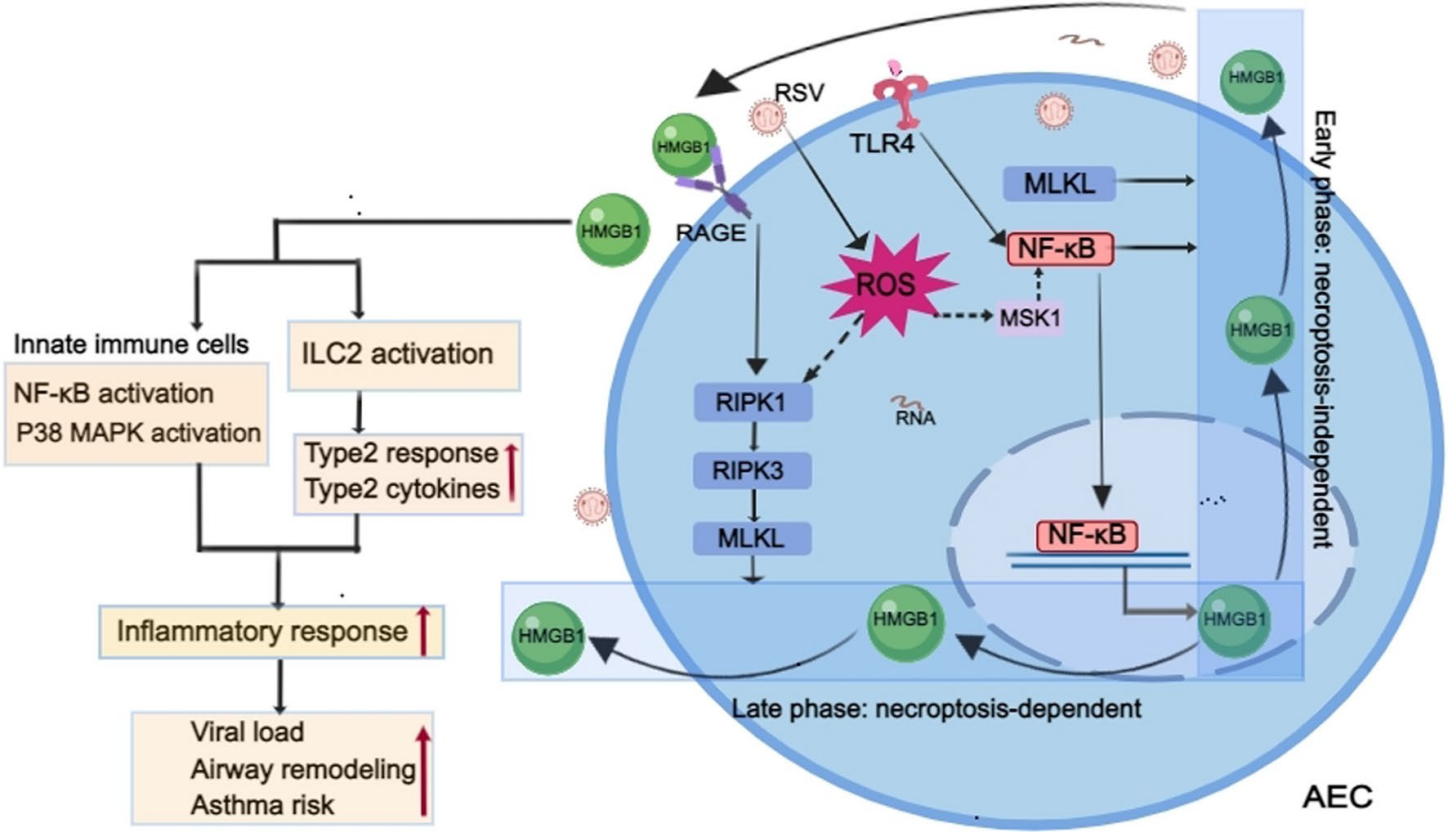
ROS-mediated HMGB1 release by AECs and HMGB1-induced excessive inflammatory response in RSV infection. RSV infection of AECs induces the biphasic release of HMGB1: early phase, which is necroptosis-independent but MLKL dependent, and late phase, which is necroptosis dependent. The early phase of HMGB1 release promotes the late phase of HMGB1 release via the activation of RAGE and initiation of RIPK1/RIPK3/MLKL phosphorylation. The TLR4/NF-κB pathway is also involved in HMGB1 release from AECs. RSV-induced ROS generation promoting the release of HMGB1 from cells likely occurs through the RIPK1/RIPK3/MLKL pathway and MSK1/NF-κB pathway. The release of HMGB1 induces an excessive inflammatory response that contributes to RSV pathogenesis

RSV infection is reported to promote HMGB1 release by AECs [97, 121]. Increased levels of HMGB1 have also been observed in the lungs of RSV-infected rat pups, peripheral blood, and nasopharyngeal aspirates in infants with RSV bronchiolitis [122–124]. Interestingly, an upregulation of the receptor for advanced glycation end products (RAGE) and HMGB1 is also found among patients with the most severe forms of COVID-19 [125], which indicates the harmful effects of augmented HMGB1 in respiratory viral infections. A study of subcellular localization indicated that RSV infection results in an overall decline in cellular HMGB1 protein levels but an enhanced translocation of HMGB1 from the nucleus to the cytoplasm and the final release into the extracellular space [121]. Oxidative stress is reported to trigger the translocation of HMGB1 from the nucleus to the extracellular space [126, 127], and antioxidants are reported to be protective in the setting of experimental infection/sepsis and injury, including ischemia‒reperfusion, partly by attenuating HMGB1 release and systemic accumulation [128]. RSV-induced ROS generation has been demonstrated to promote the release of HMGB1 from cells, and treatment with ROS scavengers has been shown to significantly inhibit HMGB1 release [121]. HMGB1 release is thought to be ROS-dependent in RSV infection. Moreover, there is another cellular event involved in HMGB1 release during RSV infection. RSV-induced epithelial cell necroptosis, characterized by increased phosphorylated RIPK1 and phosphorylated MLKL but not active caspase-3 expression, coincides with AEC sloughing, HMGB1 release, and neutrophilic inflammation [97]. Since ROS overproduction is implicated in positively regulating necroptosis of RSV-infected macrophages via the RIPK3/MLKL pathway [70], there is reason to deduce that RSV-induced ROS may also contribute to AEC necroptosis, which promotes the release of HMGB1. A recent study revealed that RSV infection of hAECs induces the biphasic release of HMGB1 at 6 (“early”) and 24 (“late”) hours post-infection (Fig. 4). The early phase of HMGB1 release at 6 h post-infection, which is cell death-independent but MLKL dependent, promotes the late phase of HMGB1 release via the activation of RAGE, initiation of a second wave of RIPK1/RIPK3/MLKL phosphorylation and occurs with cell necroptosis [129]. The TLR4/NF-κB pathway has also been confirmed to be involved in RSV-induced HMGB1 release from AECs [130]. As ROS regulate mitogen- and stress-activated protein kinase 1 (MSK1), a kinase upstream of phospho-Ser-276 formation required for RSV-induced RelA/NF-κB activation [32], it is plausible that ROS also regulate HMGB1 release through NF-κB signaling. Further research is needed to test these hypotheses.

AEC necroptosis-induced HMGB1 release is reported to be associated with increased viral load, augmented type 2 inflammation, and airway remodeling in RSV infection, and intervention to inhibit cell necroptosis can attenuate these pathologies [97]. Once activated, Group 2 innate lymphoid cells (ILC2s), which are the innate counterparts of T-helper type 2 (Th2) cells, act as potent promoters of airway inflammation and hyperresponsiveness in RSV bronchiolitis and childhood wheezing/asthma [131]. RSV-induced HMGB1 and other epithelial-derived alarmin proteins, including IL-33, IL-25, and thymic stromal lymphopoietin (TSLP), promote ILC2 activation, which leads to the induction of a type 2 response and the production of type 2 cytokines [132]. HMGB1 is mainly dependent on TLR 2,TLR 4, and RAGE to trigger intracellular MAPK and NF-κB signal transduction, and mediate innate and adoptive immune responses. It also mediates the Th2 inflammatory response with the participation of TLR3, TLR9, T-cell immunoglobulin mucin (TIM) 3, CD24, and antiN-methyl-D-aspartate receptor (NMDAR) in asthma [133]. RSV infection promotes necroptosis and HMGB1 release by AECs, which can facilitate the secretion of proinflammatory mediators and the activation of the NF-κB and P38 MAPK pathways in immune cells in a paracrine mechanism, thus promoting the inflammatory response that contributes to RSV bronchiolitis pathogenesis [97, 130]. Clinical studies have revealed that higher levels of HMGB1 correlate with the clinical severity of RSV-induced bronchiolitis [123] and act as an independent risk factor for children to develop asthma during follow-up [124]. Anti-HMGB1 mitigates both early-life viral disease and later-life asthma-like features [134]. Collectively, these results indicate that RSV-induced HMGB1 release enhances the type 2 response and the secretion of proinflammatory mediators and is associated with an elevated viral load, airway smooth muscle remodeling, and a higher risk of asthma. Therefore, blocking the proinflammatory function of HMGB1 may be an effective approach for developing novel therapeutics for RSV infection.

### ROS and DNA damage

ROS are toxic but also act as signaling factors, directly or via oxidative modifications to cellular macromolecules such as lipids, proteins, and DNA. ROS can induce DNA damage, which includes base oxidation, abasic sites, and even both single- and double-stranded DNA breaks [135, 136]. DNA double-strand breaks (DSBs) are the most dangerous type of DNA damage because they can activate DNA damage response (DDR) kinases, leading to initiation of DDR and loss of large chromosomal regions and influencing various types of DNA metabolism, especially replication and transcription. If DSBs are induced near or in transcription sites, transcription is repressed under ataxia telangiectasia mutated kinase (ATM) or DNA-dependent protein kinase catalytic subunit (DNA-PKcs) signaling [137, 138]. Programmed DNA single- or double-strand breaks seem to have a strategic role in the regulation of gene expression through the relief of DNA torsional stress and activation of promoters and enhancers [139].

**Fig. 5:**
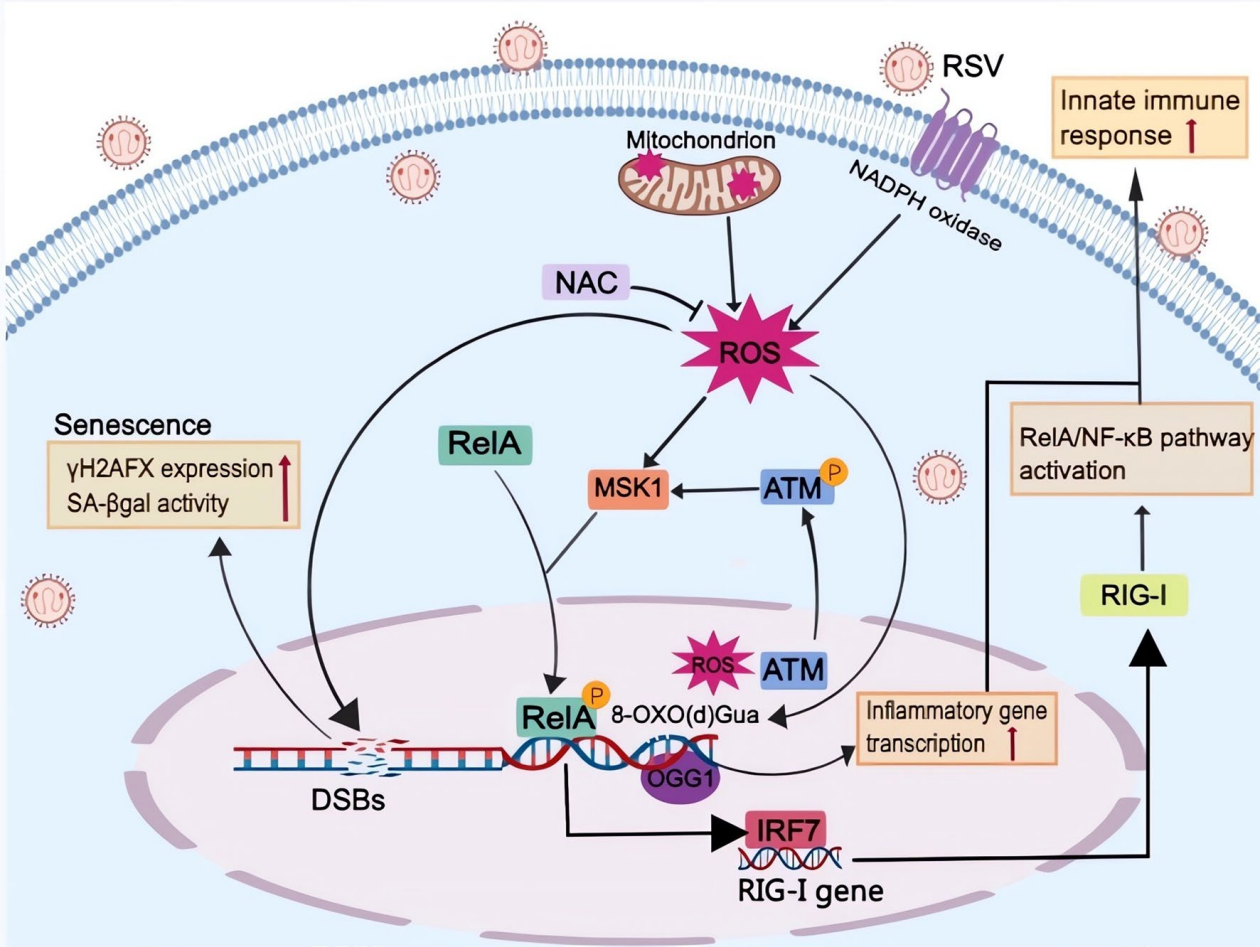
ROS-mediated DNA damage and ATM-MSK1-phospho-Ser276 RelA-IRF-RIG-I pathway in RSV-infected cells. ROS production induces DSBs and 8-oxo(d)Gua along with enrichment of enzymatically disabled OGG1 in RSV-infected cells. DSBs are associated with senescence, while OGG1 promotes the DNA occupancy of NF-κB and contributes to the augmented innate immune response. Moreover, NAC is able to reverse DSBs via the inhibition of ROS production. ATM, as a nuclear ROS and DNA damage sensor, is translocated from the nucleus into the cytosol and activates the MSK1-phospho-Ser276 RelA-IRF-RIG-I amplification loop in RSV infection

The production of ROS in cells is considered one of the most important sources of DSBs, and endogenous ROS are also able to induce the conversion of single-stranded DNA lesions into double-stranded DNA breaks [140]. Persistent DNA damage signaling triggers senescence-associated inflammatory cytokine secretion [141]. A study has shown that the production of RSV-induced mtROS results in the expression of DNA damage markers, such as phosphorylated tumor suppressor p53 (TP53), ATM, cyclin-dependent kinase inhibitor 1A (CDKN1A) and γH2AFX (H2A histone family member X, phosphorylated on Ser 139), and proliferation arrest in cultured cells (Fig. 5) [42]. ATM, as a nuclear ROS and DNA damage sensor, is activated by RSV replication and translocated from the nucleus into the cytosol. In addition, the ATM-MSK1-phospho-Ser276 RelA-IRF7- RIG-I amplification loop plays a significant role in the innate immune response to paramyxovirus virus infections, including RSV and SeV (Fig. 5) [142]. γH2AFX, a biomarker of DNA damage and aging, and tumor suppressor p53-binding protein 1 (TP53BP1), both of which are contained in DNA damage foci, indicate the existence of DSBs, the most dangerous type of DNA damage. Moreover, DSBs are associated with the accumulation of senescent cells, displaying all the hallmarks of the senescence phenotype in both mononuclear cells and syncytia [42]. RSV-induced senescence may be seen as a host defense mechanism, instructing “damaged” cells to cease proliferation. However, excessive accumulation of senescent cells may negatively affect their homeostasis because they can secrete massive amounts of factors to modulate cell growth, create a proinflammatory microenvironment or remodel the extracellular matrix [143, 144]. Thus, RSV-induced senescence may enhance airway tissue remodeling (exhibited by a loss of ciliated cells and an increase in secretory cells), potentially leading to permanent tissue damage and fibrosis [144]. The overall impact of cellular senescence on acute respiratory viruses may depend on host resilience factors [144]. Antioxidant therapy such as N-acetylcysteine (NAC) or reduced glutathione ethyl ester (GSHee) has been shown to be able to reverse DSBs [42], which may act as a protective measure to improve the pathological condition.

Generally, 7,8-dihydro-8-oxo(d)guanine (8-oxo(d)Gua) is one of the most abundant ROS-induced DNA lesions. It is removed through DNA base excision pathways by base-specific DNA repair enzymes, primarily by 8-oxoguanine DNA glycosylase 1 (OGG1) [145]. A recent study revealed that RSV infection increases the production of ROS and intrahelical 8-oxo(d)Gua primarily in transcription start site adjacent promoter sequences along with enrichment of enzymatically disabled OGG1 (Fig. 5). Since OGG1 couples RelA/NF-κB to the expression of innate immune response genes in RSV-infected lungs and cells, these interactions lead to the expression of cytokines and chemokines, prominent lung inflammation, histological changes, and body weight loss in experimental animals. Pharmacological inhibition of OGG1 significantly decreases the innate immune response and reverses the situation. This study identifies the unprecedented role of ROS-induced DNA base lesions and cognate repair proteins as determinants of RSV-induced dysregulated inflammation. Pharmaceutical inhibition of the OGG1 interaction with 8-oxo(d)Gua is expected to have clinical utility against RSV-induced excessive lung inflammation [146]. Collectively, RSV-induced ROS play an important role in DNA damage, including DNA base lesions and DSBs, which are associated with cell senescence and excessive innate immune responses.

### ROS and acquired ciliopathies

Functional motile cilia on airway cells, which serve as a necessary defense system, are critical for clearance of mucus-trapped particles and microorganisms out of the respiratory tract. Cilia contain or are quite similar to oxidant-generating systems such as nitric oxide synthases (NOS) or NADPH oxidases and mitochondria, respectively [147, 148]. Motile cilia are rich in thiol-dense and thiol-regulatory proteins that are sensitive to the local redox environment [149, 150]. Dynein ATPases drive ciliary motility and are sensitive to the local redox microenvironment in each cilium. PKA, PKC, and protein phosphatase 1, which are located in cilia and regulate cilia motility, are also redox-sensitive [147, 149–151]. Therefore, redox balance is implicated as a potential modulator of cilia motility.

Analyses of ciliated cells show the specificity and tropism of RSV, which can induce oxidative stress [35, 152, 153]. The RSV G protein CX3C motif is capable of binding to human CX3CR1, which has been demonstrated to exist on the apical surface of ciliated human AECs [154, 155]. Through engagement with CX3CR1, G protein is reported to facilitate attachment and viral penetration into the host cell that results in nucleolin expression as well as suppression of gene transcripts specific to ciliated cells, which is coincident with the decreases in ciliated cells [155, 156]. However, another study has shown a different outcome. In this study, RNA sequencing (RNA-seq) was performed in nasal scrape biopsies obtained from infants admitted to the pediatric intensive care unit with critical RSV bronchiolitis requiring invasive or noninvasive respiratory support. The results revealed that increased expression of ciliated cell genes and estimated ciliated cell abundance positively correlate with the duration of hospitalization in infants with critical bronchiolitis [157]. One possible explanation of the discrepancy may be the different cell sources and distinguishing severity of RSV infection. The presence of the F protein in the cilia is also reported to contribute to cellular changes in the cilia and reduced cilia function [158]. Increased ciliary dyskinesia combined with ciliary loss and epithelial damage is an early feature of RSV infection, which is likely to result in reduced mucociliary clearance. Neither reduced ciliary beat frequency (CBF) nor reduced motile ciliated cells but rather an increased number of cilia with an abnormal beat pattern were observed in RSV-infected human ciliated nasal epithelial cells [153]. However, another study reported that RSV infection decreases CBF and affects mucociliary clearance at 4 days post infection [159]. A recent study has shown that RSV infection blocks cilia beating from day four, while human metapneumovirus infection does not affect cilia beating [160]. All of these results suggest that RSV infection affects the expression of ciliated cell genes, the number of cilia, ciliary dyskinesia and ciliary loss.

**Fig. 6:**
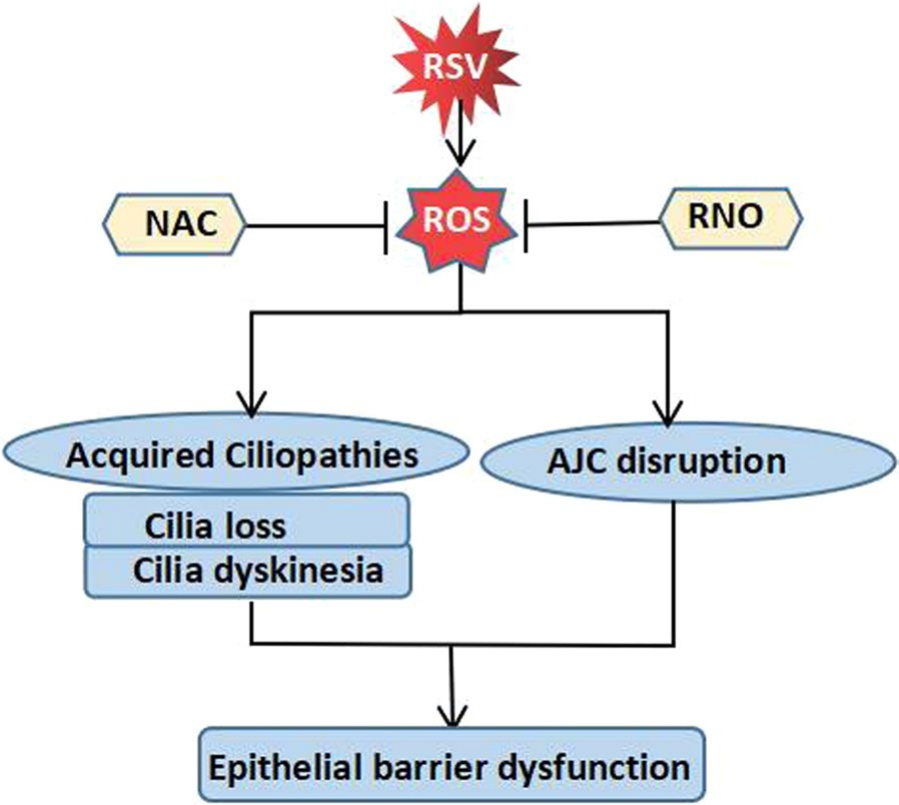
ROS-mediated acquired ciliopathies and barrier dysfunctions in RSV infection. RSV-induced ROS are thought to play a significant role in cilia loss and dysfunction as well as AJC disruption, which result in epithelial barrier dysfunction and can be blocked by NAC or RNO by reducing ROS production

Mata et al. reported that RSV-induced H_2_O_2_ plays a significant role in cilia loss and dysfunction. Preincubation with NAC improves the situation by decreasing H_2_O_2_ generation and promoting the expression of GSH, Nrf2 and heme oxygenase 1 (HO-1), which enhances the antioxidant effect, reverses the RSV-induced loss of ciliogenesis and decreases ciliary activity in an in vitro model of RSV infection in human bronchial epithelial cells [161]. Roflumilast N-oxide (RNO), a phosphodiesterase 4 (PDE4) inhibitor, also restores cilia motility and reverses a loss in cilia following RSV infection in human bronchial epithelial cells. In this process, RNO has been demonstrated to support the antioxidative apparatus compromised in RSV-infected cells and reduce ROS [162]. In addition, excessive production of RSV-induced ROS results in barrier dysfunction, which is characterized by apical junctional complex (AJC) disruption and can be exaggerated by titanium dioxide nanoparticles (TiO2-NPs) and reversed by antioxidants [163]. The effects of these interventions indicate that ROS and oxidative stress may contribute to “acquired ciliopathies” and barrier dysfunctions, and antioxidative treatment may be a potential measure to improve the situation (Fig. 6) [150].

## Conclusions

The redox balance between pro-oxidants and antioxidants might directly or indirectly affect the progression and outcome of viral infections [164]. In this review, we emphasized the oxidative stress and ROS-mediated cellular events in RSV infection. Although proper ROS production serves as a beneficial host defense response, RSV-induced ROS overproduction plays an important role in many cellular events, including the elevated release of HMGB1 and NETs, the activation of NETosis and the NLRP3 inflammasome, increased DNA damage, “acquired ciliopathies” and barrier damage. Most of these ROS-mediated cellular events seem to do more harm than good in RSV infection. Substantial evidence has shown the beneficial effects of antioxidant treatment in severe RSV infection, such as inhibition of RSV replication and improvement of barrier dysfunction and lung inflammatory injury [15, 50, 62, 163, 165]. In addition, zinc is a stable divalent cation and does not directly undergo redox reactions, but it is involved in regulating the oxidant/antioxidant balance [166]. During RSV infection in A549 cells, both zinc depletion and the addition of exogenous ROS are beneficial for virus replication, while adding zinc blocks replication. Zinc chelation leads to ROS induction, whereas the addition of zinc blocks ROS induction [35]. All of these results indicate antioxidant treatment as a promising and novel approach to ameliorate RSV-induced acute lung inflammation and potentially prevent long-term consequences associated with RSV infection. However, there is still a pressing need to further characterize pathways, including those generated by oxidative stress, to prove the effect of antioxidant treatment and determine more effective therapeutic measures to improve the outcome of RSV infection (Table 1).

**Table 1:** The ROS-mediated cellular events and related main adverse effects in severe RSV infection

The ROS-mediated cellular events	The main adverse effects
NLRP3 inflammasome activation	Excessive innate immune responses and lung inflammatory injury
NET release	Airway obstruction, mucus hypersecretion, and heightened lung damage
HMGB1 release	Increased viral load, augmented type 2 inflammation, and airway remodeling
DNA damage	Excessive innate immune responses, cell senescence, and airway tissue remodeling
Acquired ciliopathies	Barrier dysfunctions and reduced mucociliary clearance

## Data Availability

Not applicable.

## Abbreviations

AJC: Apical junctional complex
ALI: Acute lung injury
AOEs: Antioxidant enzymes
ARDS: Acute respiratory distress syndrome
AREs: Antioxidant response elements
ASC: Apoptosis-associated speck-like protein containing a caspase recruitment domain
ATM: Ataxia telangiectasia mutated kinase
CBF: Ciliary beat frequency
CDKN1A: Cyclin-dependent kinase inhibitor 1A
CLRs: C-type lectin receptors
DAMPs: Damage-associated molecular patterns
DDR: DNA damage response
DNA: Deoxyribonucleic acid
DPI: Diphenyleneiodonium
DSBs: Double-strand breaks
ER: Endoplasmic reticulum
ERK1/2: Extracellular signal-regulated protein kinases 1 and 2
F: Fusion protein
FDA: Food and Drug Administration
G: Attachment protein
GSHee: Glutathione ethyl ester
H2O2: Hydrogen peroxide
hAECs: Human airway epithelial cells
HMGB1: High-mobility group box 1
HO^·^: Hydroxyl radical
HO-1: Hemeoxygenase 1
IFNRs: Interferon receptors
ILC2: Group 2 innate lymphoid cells
IRAK1: Interleukin-1 receptor-associated kinase 1
IRF: Interferon regulatory factor
IRG1: Immune-responsive gene-1
Keap1: Kelch-like ECH-associated protein 1
LAIR: Leukocyte-associated immunoglobulin-like receptor
LRTI: Lower respiratory tract infection
mAb: Monoclonal antibody
Maf: Musculoaponeurotic fibrosarcoma oncogene homolog
MAPK: Mitogen-activated protein kinases
MitoQ: Mitoquinone mesylate
MLKL: Mixed lineage kinase domain-like pseudokinase
MPO: Myeloperoxidase
MSK1: Mitogen- and stress-activated protein kinase 1
mt-Ca^2+^: Mitochondrial lumen Ca^2+^
mtROS: Mitochondrial ROS
MyD88: Myeloid differentiation primary response 88
NAC: *N*-Acetylcysteine
NADPH Oxidases/NOX: Nicotinamide adenine dinucleotide phosphate oxidases
NE: Neutrophil elastase
NET: Neutrophil extracellular trap
NF-κB: Nuclear factor kappa-light-chain-enhancer of activated B cells
NLRs: Nod-like receptors
NLRP: Nod-like receptor protein
NMDAR: *N*-Methyl-d-aspartate receptor
NOS: Nitric oxide synthase
Nrf2: Nuclear factor erythroid 2-related factor 2
O_2_^−^: Superoxide anion
OGG1: 8-Oxoguanine DNA glycosylase1
PAD4: Peptidyl arginase deaminase 4
PAMPs: Pathogen-associated molecular patterns
PDE4: Phosphodiesterase 4
PI3K: Phosphatidylinositol 3-kinase
PKC: Protein kinase C
IL: Interleukin
PRRs: Pattern recognition receptors
RAGE: Receptor for advanced glycation end products
RIG-I: Retinoic acid-inducible gene-I
RIPK3: Receptor-interacting serine/threonine-protein kinase 3
RLRs: RIG-I-like receptors
RNO: Roflumilast N-oxide
ROS: Reactive oxygen species
RSV: Respiratory syncytial virus
RSVpreF: RSV prefusion F protein-based
SARS-CoV-2: Severe acute respiratory syndrome coronavirus 2
SIRL: Signal inhibitory receptor on leukocytes
SOD 1: Superoxide dismutase 1
Th2: T helper type 2
TIM 3: T-cell immunoglobulin mucin 3
TiO2-NP: Titanium dioxide nanoparticles
TLRs: Toll-like receptors
TNFRs: Tumor necrosis factor receptors
TP53: Tumor suppressor p53
TP53BP1: Tumor suppressor p53-binding protein 1
TRAF6: TNF receptor-associated factor 6
TRPM2: Transient receptor potential melastatin 2
TSLP: Thymic stromal lymphopoietin
TXNIP: Thioredoxin-interacting protein
UPR: Unfolded-protein response
XO: Xanthine oxidase
γH2AFX: H2A histone family member X, phosphorylated on ser 139
8-oxo(d)guanine: 8-Oxo(d)Gua
